# Myxolipoma of hand in a child: case report of a rare tumor

**DOI:** 10.1007/s40477-022-00727-7

**Published:** 2022-09-24

**Authors:** Jeena Bordoloi Deka, Mohit Veer Kumar Shah, Ritu Shah, Nidhi Bhatnagar, Chandra Bortolotto, Fernando Jiménez

**Affiliations:** 1 Dispur Polyclinic and Hospitals Pvt Ltd. Guwahati, Guwahati, India; 2 Abhipraay, Centre for Advance Ultrasound Guided Interventions and Genetic Clinic, Mumbai, India; 3 Seth G S Medical College and King Edward Memorial Hospital, Mumbai, India; 4 Heading Radiology Department, Mata Devi Hospital Max Hospital, Panchsheel, New Delhi, India; 5 Radiology Unit, Università di Pavia, Pavia, Italy; 6 Sport Sciences Faculty, Castilla la Mancha University, Toledo, Spain; 7 Director of MSK US International Chair, UCAM, Murcia, Spain; 8 MSK US International Chair, UCAM, Murcia, Spain; 9 Radiology Department, Fondazione IRCCS Policlinico San Matteo, Viale Golgi 19, Pavia, Italy

**Keywords:** Myxolipoma, Hand, Pediatric, Ultrasound

## Abstract

Lipomatous tumors account for less than 10% of tumors in the pediatric population. Myxolipomas (a subset of lipoma characterised by mature adipose tissue and abundant mucoid substance) are found to be even rarer. There are a few case reports in different body parts like heart, kidney, oral cavity, epiglottis, cervical and mediastinal regions. However, there are no case reports on the involvement of the hands in any age group. High resolution ultrasound is the imaging modality of choice for the initial evaluation of superficial soft tissue tumors, their site, nature and extent. In conjunction with clinical findings and age of presentation, it helps in narrowing down the differential diagnosis and planning the management. Hyperechoic fatty tumors in the pediatric hand are mostly benign and includes lipomas, lipoblastomas and fibrous hamartomas of infancy as the main differentials. A definitive diagnosis is based on a histo-pathological and molecular cytogenetic examination. This article presents a never before reported case of a rare, large, myxolipoma of the hand in a 22-month-old boy.

## Introduction

Lipomatous tumors account for less than 10% of tumors in the pediatric population. There are several variants of lipomas based on the type of mesenchymal components present and the histopathological findings.

Myxolipoma is one such uncommon variant characterised by mature adipose tissue and abundant mucoid substance.

High resolution musculoskeletal ultrasound is the initial imaging modality of choice for superficial soft tissue tumors in children. Key imaging features of lipomatous tumors, in conjunction with clinical findings, age of presentation and site of involvement help to narrow down the differential diagnosis. A definitive diagnosis is based on a histopathological examination.

We present a case of a very large myxolipoma in the subcutaneous tissue of the hand in a 22-month old boy.

There have been very few case reports of myxolipoma in various parts of the body such as the heart, kidney, oral cavity, cervical and mediastinal regions.

However, after an extensive review of relevant literature, we believe that this is the youngest case report of myxolipoma anywhere, as well as the only one that has been reported in the hand, making it a unique case.

## Case presentation

**Fig. 1:**
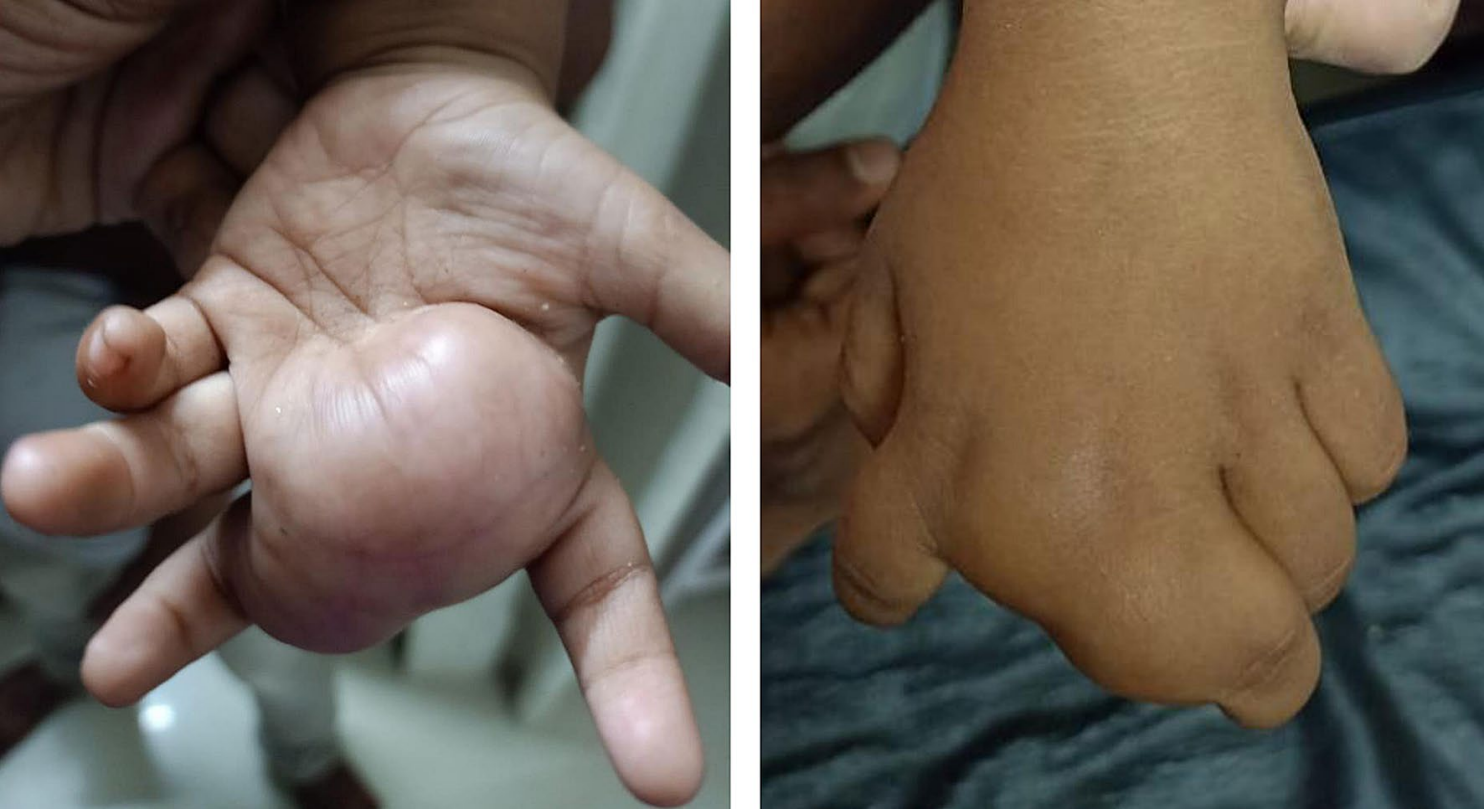
The clinical photos shows the large soft tissue mass widening the second and third webspace in the palmar aspect (**a**) and the extension of the lesion to the proximal phalanx of the 2nd finger in the dorsal aspect (**b**)

A 22 month old male presented with a painless swelling in the left hand, that caused difficulty in grasping objects. The swelling was predominantly in the palmar aspect causing widening of the 2nd and 3rd web space (Fig. 1). It was first noticed by the parents 4 months ago, and has since grown in size. It measured approximately 40 × 30 mm in dimensions. The lesion extended upto the proximal phalanx of the 2nd digit. The overlying skin was normal in color and texture. On palpation, the lesion was nonmobile, non-pulsatile and firm to touch.

### Investigations

**Fig. 2:**
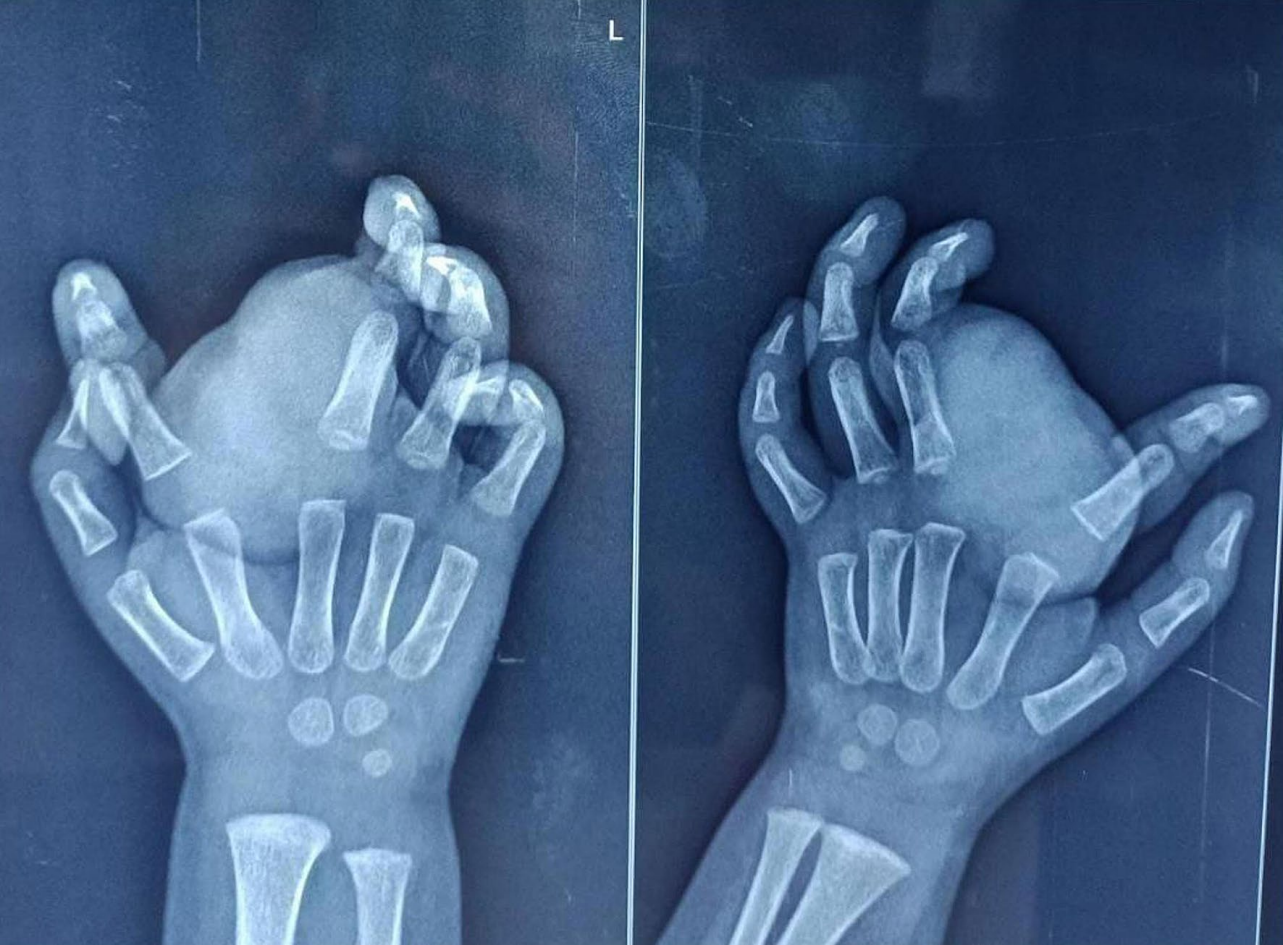
Plain X ray AP (**a**) and oblique views (**b**) of the left hand. Homogenous soft tissue mass with a smooth outline and lobulated contour in the region of the 2nd web space, widening the space between the 2nd and 3rd metacarpals and phalanges. Bones are normal

**Fig. 3:**
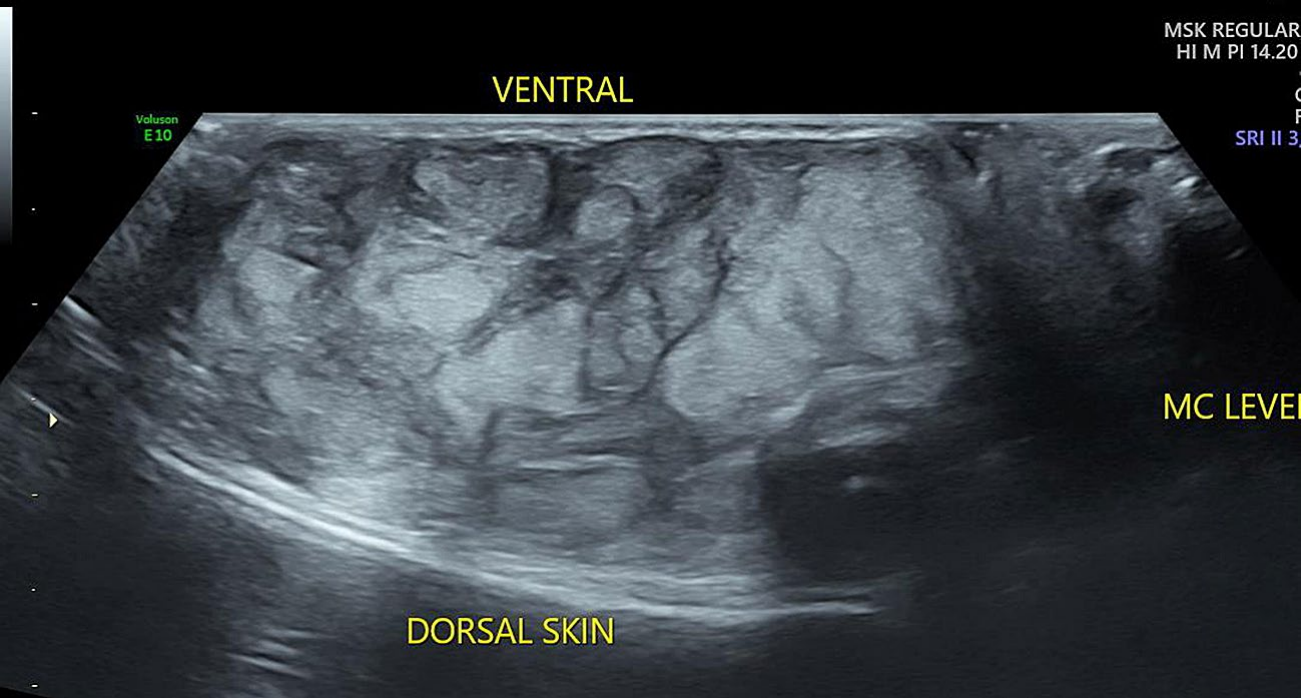
Gray scale US image in long axis in the 2nd intermetacarpal space shows a well defined heterogenous predominantly echogenic mass (M) in the subcutaneous soft tissues of the hand extending to the level of the proximal phalanx and distal metacarpal (MC). Mass has a lobulated contour and extends from the ventral to the dorsal skin. Intervening hypoechoic areas are seen within lobulated hyperechoic regions

**Fig. 4:**
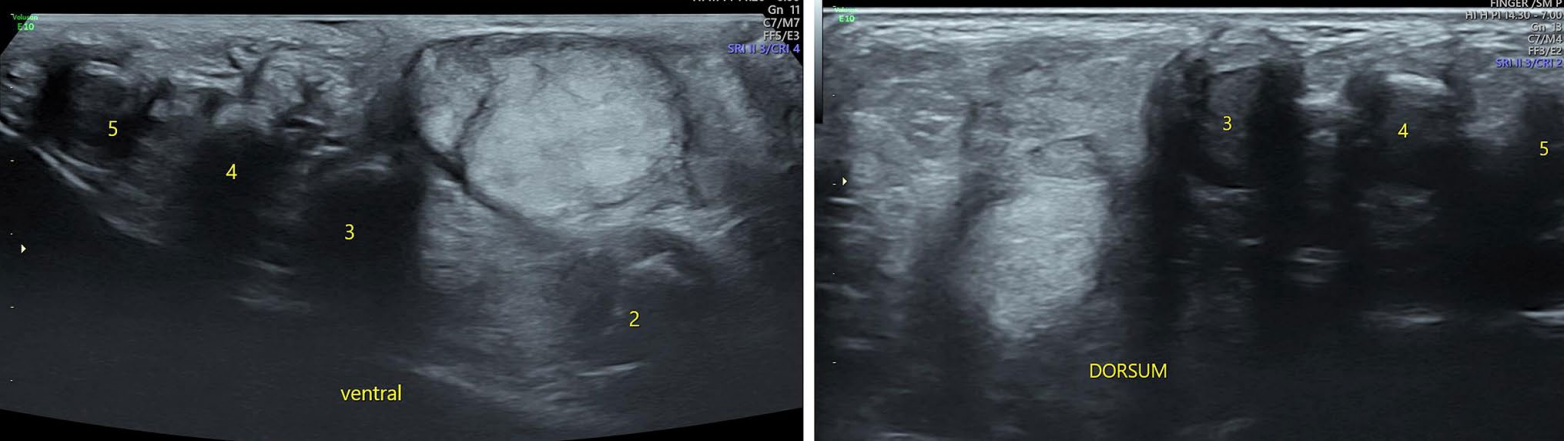
**a** and **b** Gray scale US images in short axis seen from ventral (**a**) and dorsal aspects (**b**) at the level of distal shaft of metacarpals reveals the mass (M) in between the 2nd and 3rd metacarpals. A hypoechoic plane of cleavage separates it from the underlying muscles and tendons

**Fig. 5:**
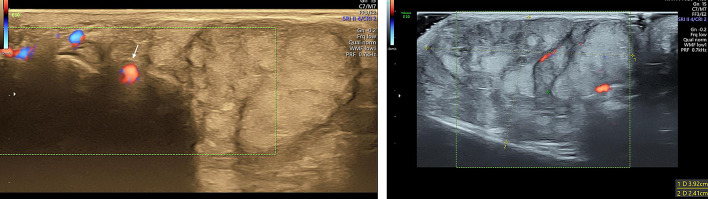
**a** and **b** Doppler ultrasound with power doppler shows minimal vascularity within the Mass (M). The 2nd interdigital artery (arrow) is seen away from the mass

X-ray and high resolution ultrasound were advised to determine nature and extent of lesion. Plain X-ray of the hand revealed a lobulated homogeneous, smoothly outlined, soft tissue mass which was seen to cause widening of the webspace between the 2nd and 3rd fingers. The underlying bones were normal with no evidence of destruction, erosion or sclerosis (Fig. 2). The child was examined with a high resolution (ML 6–15) linear probe in a Voluson E10 (GE Healthcare, Austria) ultrasound machine. On gray scale imaging, a well-defined, heterogenous, predominantly echogenic mass with a lobulated contour was seen in the subcutaneous soft tissues of the palmar aspect of the left hand mainly involving the 2nd and 3rd web space. It was seen extending to the proximal phalanx of the 2nd digit dorsally. Intervening linear hypoechoic areas were seen within lobulated hyperechoic regions. (Fig. 3). The lesion measured approximately 40 × 25 mm in size and in the deeper part was seen adjoining the flexor tendons of the of the 2nd and 3rd fingers (Fig. 4). Dynamic scanning revealed that there was no involvement of the underlying muscles and tendons. Color Doppler study with power doppler showed minimal vascularization. The adjacent digital arteries were splayed by the lesion (Fig. 5). Imaging features, along with age of occurrence and clinical features of an insidious, slow growing, painless tumor suggested a fat containing benign tumor.

Differential diagnosis given were lipoma, lipoblastoma and fibrous hamartoma of infancy.

Biopsy was suggested for a definitive diagnosis.

### Treatment

**Fig. 6:**
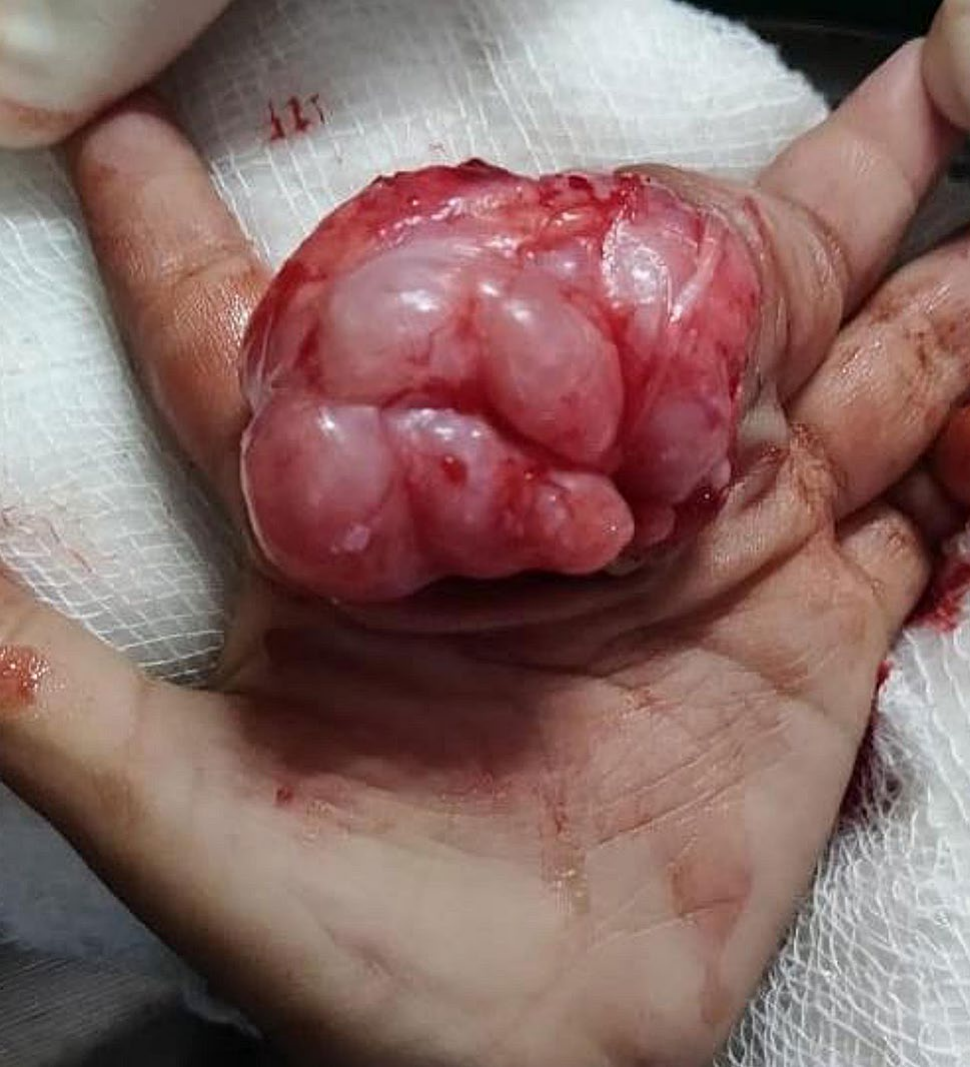
Per-operative picture reveals a well circumscribed multilobulated soft tissue mass lesion exposed as soon as the incision was made in the 2nd web space

**Fig. 7:**
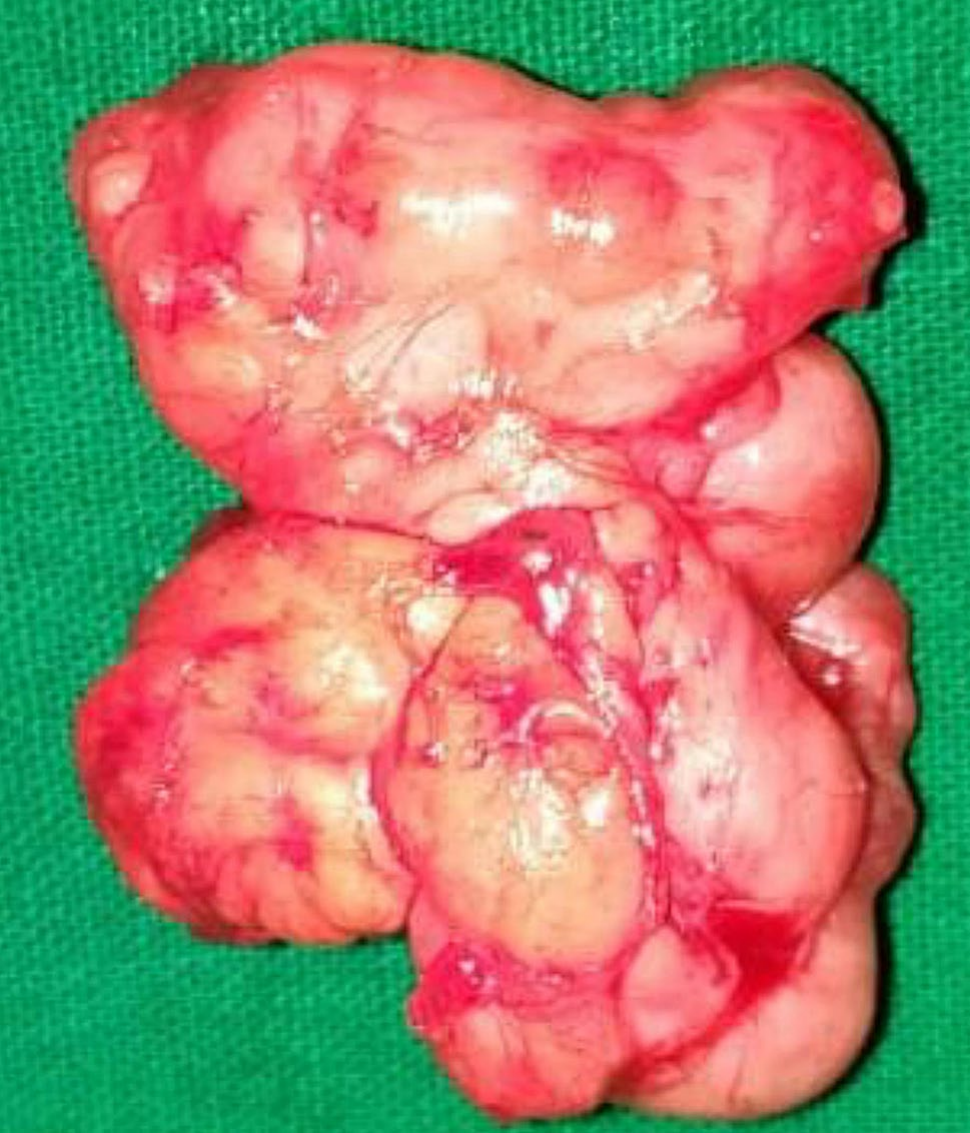
Gross macroscopic specimen reveals a lobulated yellowish mass suggestive of fatty component

**Fig. 8:**
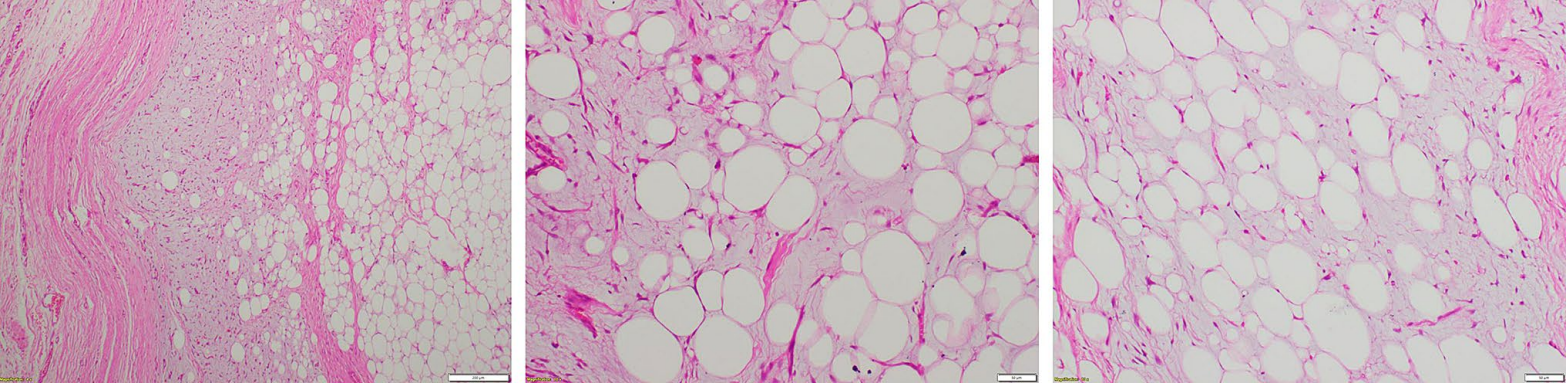
Microscopic examination with Hemotoxylin and Eosin staining reveals lobules of mature adipose tissue, extensive myxoid change in the stroma with a network of interspersed thin walled blood vessels

The parents agreed to an excision biopsy and the entire tumor was removed surgically (Fig. 6). Gross examination of the specimen revealed a yellowish and gray white soft tissue lesion of size 40 × 30 × 25 mm and showed a lobulated contour (Fig. 7). Cut section showed soft to mucoid areas. Microscopic examination showed a well circumscribed lesion composed of lobules of mature adipose tissue along with a network of interspersed thin walled blood vessels. The stroma showed extensive myxoid change. No inflammation, lipoblasts or atypia was seen (Fig. 8). The findings were consistent with a myxolipoma of the hand.

On follow up after 3 months, the patient showed greater mobility of his fingers and was able to grasp objects. No recurrence was reported in the 12 months follow up.

## Discussion

Adipocytic tumours are mesenchymal tumors with considerable morphologic and genetic heterogenicity [1]. In children,adipose and myxoid tumors form a challenging group of neoplasms in contrast to adults [2]. Lipomatous tumors,which is common in adults,account for less than 10% of all soft tissue lesions in pediatric patients [3]. Out of them, lipomas are benign mesenchymal tumors which comprise of only 4% of all soft tissue tumors in children [3–5]. Myxolipoma is one of the rare histological varieties, accounting for less than < 1% of lipomas [6].

There is no mention of myxolipoma in the WHO classification of tumors of Soft tissue and Bones (2013). A simplified classification of myxoid soft tissue tumors based on this WHO classification was given by Baheti et al. [7]. They have classified myxolipoma to be a rare benign myxoid soft tissue neoplasm. Coffin et al. [2] have also mentioned myxoid lipoma to be benign myxoid tumor of soft tissue in children and adolescents. There have been few case reports of myxolipomas in different parts of body like oral cavity, cervico-mediastinal region, retroperitoneum, heart, tongue, epiglottis and kidney. These have been reported in adults and in children above 5 years [6, 8, 9].

High resolution Musculoskeletal Ultrasound is the preferred imaging modality for evaluation of superficial soft tissue masses in all age groups and is the initial choice in pediatric population [5, 10]. It is important not to distort the lesion by applying too much pressure and hence applying sufficient amount of gel is advised. The unifying ultrasound feature of hyperechoic subcutaneous soft tissue masses tumors is the presence of fat which corresponds pathologically to adipose tissue [11]. Fat admixed with other soft tissue components are more echogenic because of the increased reflectivity of fat from different interfaces [5, 10].

Majority of hyperechoic fat containing tumors involving the hand in children are adipocytic tumors like lipomas, lipoblastomas and liposarcomas. Fat may also be present in non adipocytic fibroblastic/myofibroblastic tumors like fibrous hamartoma of infancy and lipofibromatosis which should also be considered in the differential diagnosis [10].

The presence of intralesional fat in soft tissue hand tumors in children suggests a benign etiology [2, 10]. The primary exception is a liposarcoma [11].

Lipoblastomas are hyperechoic fatty mass with well defined margins, predominantly seen in children less than 3 years of age. They can have myxoid fatty components that can predominate. [12].

Liposarcomas are malignant soft tissue sarcomas, hyperechoic solid mass without cystic components and variable vascularity [11]. They are rarely seen in children less than 10 years age and are more infiltrative [3, 10]. Fibrous hamartoma of infancy display hyperechoic areas with hypoechoic zones showing “serpentine pattern” or predominantly trabeculated hypoechoic bands and a peripheral halo. Doppler shows poor vascularity [5]. They occur in the first 2 years of life and can involve both upper and lower extremities and hands [13].

Lipofibromatosis are hyperechoic lesions without cystic components and variable vascularity [10]. They are seen in infancy and childhood with a predilection for hands and feet.

There have been case reports of Histological variants like Angiomyxolipoma in the feet and spindle shaped lipoma of dorsum of the hand in the feet and dorsum of the hand in children [14, 15].

In all cases, a biopsy is usually required for a definitive diagnosis [16]. Minimally invasive biopsies have become more common now. Pathologic examination is enhanced by adjunct techniques like cytogenetic or molecular genetic studies, further refining the pathologic classifications [1, 2]. Molecular markers help in proper characterization of fat containing soft tissue tumors [11]. Ability to identify chromosomal abnormalities has been markedly improved by development of molecular cytogenetic technologies like fluorosence insitu hybridization (FISH). Immunohistochemistry may have only a little role (17).

In the present case, the ultrasound appearance of a hyperechoic well-defined mass with intervening hypoechoic areas suggested a lipomatous tumor admixed with some our mesenchymal component. Dynamic study helped in evaluating extent as well as involvement of adjacent muscles and tendons. Color Doppler helped in evaluating the vascularity. Excision biopsy confirmed the lesion to be myxolipoma of the hand.

### Learning points/take home message/conclusion

Myxolipoma, a variant of lipoma, is a rare benign adipose tumor that can now be considered as a differential diagnosis in lipomatous soft tissue tumors of the extremities in all age groups and in the pediatric hand. A biopsy is imperative for a final histo-pathological diagnosis. High resolution musculoskeletal ultrasound with dynamic study in pediatric hand tumors is important as an initial imaging modality of choice. It helps to evaluate the nature of the lesion, predominant mesenchymal composition, vascularity, extent, and relationship with adjacent structures. Used in conjunction with clinical findings like age, progress and site of involvement, ultrasound helps in narrowing the differential diagnosis, gives an idea about the benign or malignant nature of the lesion and in planning the management.
